# Data Policy and Data Sharing Agreement in the LifeWatchGreece Research Infrastructure

**DOI:** 10.3897/BDJ.4.e10849

**Published:** 2016-11-01

**Authors:** Eva Chatzinikolaou, Sarah Faulwetter, Dimitra Mavraki, Tilemachos Bourtzis, Christos Arvanitidis

**Affiliations:** ‡ Institute of Marine Biology, Biotechnology and Aquaculture, Hellenic Centre for Marine Research, Heraklion, Crete, Greece; § University of Patras, Department of Biology, Laboratory of Zoology, Rio, Patras, Greece; | European Centre for Environmental Research and Training, Panteion University, Athens, Greece

## Abstract

The LifeWatchGreece Research Infrastructure (LWG RI) stores biodiversity data and information from all biology-related disciplines derived from the Greek territory (or the Mediterranean Sea for the marine data). The aim of LWG RI is to facilitate data sharing and dissemination under harmonised standards in order to maximize the socio-economic benefits of research and knowledge transfer to the public. This publication describes the rationale behind the data policy of LWG RI, outlines the current legal situation for sharing research data and presents the Data Sharing Agreement which is signed between the data owner/provider and the LWG RI for each dataset, describing in detail the rights and duties of each party, as well as the license type and the embargo period under which the data are released.

## Keywords

LifeWatch, Biodiversity data, Greece, Copyright, Data policy, Data sharing, Data licenses, Agreement, Rights and duties

## Introduction

Greece hosts a significant biological diversity, at molecular (gene), species and ecosystem level. This is a result of its geological and evolutionary history, its complex geomorphology and topography, the relatively mild human pressures received until recently, and its range of 29 climatic classes (according to the Thornthwaite classification scheme; (Ministry of Environment, Energy and Climate Change 2014)). According to the Annex I - Directive 92/43/EEC classification scheme, there are 25 habitat types in Greece, including maquis, phrygana, forests, grasslands, marine areas, wetlands, lagoons, salt marshes and sandy beaches. With the longest coastline in the Mediterranean (13,676 km as retrieved from the CIA World Factbook in September 2016), marine and coastal habitats are the largest of these. Although most probably the largest part of Greek biodiversity remains non-described (Legakis 2015), existing data clearly indicate that it is particularly rich regarding wild fauna and flora, as well as genetic resources related to agriculture and food products (Ministry of Environment, Energy and Climate Change 2014). The most recent available numbers show that up to 23,130 animal species have been recorded in the terrestrial and freshwater ecosystems of Greece (Legakis and Maragou 2009, [Bibr B3437202][Bibr B3437202], Legakis and Maragou 2009), of which 3,956 are characterised as endemic (Fauna Europaea 2004). Furthermore, 3,500 animal species have been recorded in the Greek marine environment. The Greek flora includes 5,752 species of which 1,278 are endemic (i.e. 22.2% of all species present) (Georgiou and Delipetrou 2010). Consequently, Greece can be considered as a hot spot area for the European and the Mediterranean biological diversity, because it is characterized by a high level of endemism and according to its Convention of Biological Diversity profile it comprises one of the last refuges of many threatened, endangered and rare species on a European scale. Therefore, the effective protection of biodiversity in Greece, which constitutes the nation's natural capital, requires the collection, archiving and free accessibility of information and accurate scientific data, which only a national open Research Infrastructure, as part of a broader European Infrastructure, could guarantee.

## What is the LifeWatchGreece Research Infrastructure

The European e-Science Research Infrastructure for biodiversity and ecosystem research, LifeWatch, is designed to provide advanced research and innovation capabilities on the complex biodiversity domain. A Research Infrastructure (RI) refers to the strategic installation of facilities, resources and related services for the benefit of the scientific and other user's communities (Commission Staff Working Document 2013). An e-Science infrastructure can capitalize existing resources, as well as data and data observatories from physical infrastructures, distributed centers and single research groups. The LifeWatchGreece Research Infrastructure (LWG RI) is the national node of LifeWatch in Greece, which has been constructed and operated by the Hellenic Centre for Marine Research at the Institute of Marine Biology, Biotechnology and Aquaculture and aims to establish a Centre of Excellence for biodiversity research in the Southeastern Europe. The LWG RI stores biodiversity data and information from all biology-related disciplines and it is open to all potential users, such as the scientific and academic community, environmental managers, policy makers, students and citizen scientists. The purpose of LWG RI is to accelerate international data-driven innovation and discovery by facilitating data sharing and exchange, data use and re-use, harmonization of standards and discoverability.

The ultimate goal of LWG RI is based on the European Strategy Forum on Research Infrastructures (ESFRI) policy on research and innovation RIs, which seeks to maximize the socio-economic benefits of research and development for the public. According to the Communication from the Commission to the European Parliament, the Council and the European Economic and Social Committee on “Scientific information in the digital age: access, dissemination and preservation ({SEC(2007)181}/*COM/2007/0056 final): "*All research builds on former work, and depends on scientists’ possibilities to access and share scientific publications and research data. The rapid and widespread dissemination of research results can help accelerate innovation and avoid duplication of research efforts*”. The European Commission supports open access as the standard way of disseminating publicly funded research in the European Union and includes open circulation of knowledge as one of the five priorities of the European Research Area. Open access is required for all the peer-reviewed publications resulting from Horizon 2020 funding. Recent EU activity on data policy shows that future public funded research will focus on open access requirements, making the adoption of such policies essential both for the sustainability of the LWG RI, as well as for the participating researchers. Under this context, LWG RI seeks to aggregate and disseminate biodiversity data according to the RDA principles of non-profit, openness, consensus-based decision making, balanced representation, technical neutrality and harmonization across communities and technologies (RDA 2014).

## Data Sharing and Open Access: why should I share my data?

Data are individual pieces of information which can form a dataset when they are thematically related and systematically organised. They are a critical and irreplaceable resource, particularly for the assessment, monitoring, protection and sustainable management of biodiversity and of the resulting direct and indirect economic benefits. Biodiversity and ecosystem data have a thematical and historical value since they document the status of a dynamic system at a specific point in space and time and are thus irreproducible (Costello 2009, Costello et al. 2013, Hardisty, A. et al. 2013). A financial value can also be assigned to such data in terms of staff time, ship/sampling time and use of equipment. Therefore, re-use of data is cost-efficient and can prevent the duplication of effort and the waste of money (Heidorn 2008, Hardisty, A. et al. 2013). Data can also have unforeseen added values beyond their initial purpose, when re-combined with other data and analysed in a different context (Costello 2009). In addition, there is a strong argument that publicly funded research data should be accessible to all tax payers and be able to survive beyond the scientist’s life-cycle in order to foster future development (Enke et al. 2012).

Milestone definitions of open access data include those of the Budapest Open Access Initiative (BOAI), the Berlin Declaration (October 2003) and the Bouchout Declaration for Open Biodiversity Knowledge Management (2014). Sharing of data comes with a number of benefits for the scientific community and the data provider:

1. Data are securely archived and stored in a format which ensures their readability in the future.
2. Data are quality controlled concerning taxonomy, geography and general integrity.
3. Datasets are fully documented with the appropriate metadata so that they can be easily located and retrieved through online search engines. These metadata are "data about the dataset" and they consist of a minimum set of information describing the scope, purpose, content, involved persons and the terms of use (licenses and embargo period) of the actual dataset.
4. Data are disseminated through major data aggregators, ensuring a global and perpetual visibility, and thus promoting the scientist's work and increasing their potential for attracting collaborations and funding.
5. Shared data are citable, giving due credit for each use of the dataset to the data provider. Downloading metrics will allow scientists to assess how often their data are viewed/ downloaded.
6. Re-use of data in a wider context can provide new insights; small datasets from projects, when integrated, can answer global questions.

Open access of data can address the problem of limited access to scientific research information (articles, monographs, research data, etc.) so that they can be further used and exploited by researchers, industry, relevant markets, and the general society (Costello 2009). Publishers and data providers who adopt open access will obtain more exposure for their publications and can focus on providing new added value services to their community (Costello 2009). Small and Medium Enterprises (SMEs) can take advantage from open access scientific data in order to create innovative products, increase their competitiveness and create job opportunities (MedOANet 2013).

## ﻿Types﻿ of dat﻿a that can be submitted to the LifeWatchGreece repo﻿sitory

According to Egloff et al. (2014) the principles of open access should be applied to every form of scientific knowledge, such as raw or processed data, metadata, source materials, as well as figures and graphs derived from these data. Thus, The LifeWatchGreece RI has created a data infrastructure that can accept and integrate all kinds of biodiversity-related data, such as:

- Species occurrence data: marine distribution data (if at least one of the distribution points in the dataset falls within the boundaries of the Mediterranean Sea) and terrestrial distribution data (if at least one of the distribution points in the dataset falls in the Greek territory)
- Taxonomic checklists
- Genetic data
- Genomics data (including meta-barcoding and metagenomics data)
- Protein data (including those resulting from proteomics)
- Morphometric and three-dimensional morphological data
- Functional trait data
- Habitat maps
- Museum collection data: any data on specimens that were collected within the boundaries of the Greek territory or are held within Greek Natural History Museums
- Environmental data: marine environmental data (only when accompanying species data, since other repositories, such as SeaDataNet and EMODnet, already exist for the submission of marine physiochemical data) and terrestrial environmental data (can be stored in LifeWatchGreece RI even without associated species data if no other repository for environmental data exists)
- Metadata (information about datasets only)
- Literature
- Sound, video, photos, 3-D mapping of environments
- Citizen Science data

In case of submission of a non-Greek dataset (or non-Mediterranean in the case of marine data) collected or owned by a researcher associated with a Greek institution, the LifeWatchGreece RI data managers will submit the data to the appropriate repository (if this exists) and store the link as an external dataset together with the metadata. If no such appropriate external repository exists, the data will be stored within LifeWatchGreece RI, without being available by default through its portal.

## Copyrights and Creative Commons licenses

Intellectual property (IP) rights are the legally recognized exclusive rights to creations of the mind (WIPO 2011). Under intellectual property law, owners are granted certain exclusive rights to a variety of intangible assets, such as literary works, discoveries and inventions (Carpenter and Dunung 2011). Common types of intellectual property rights include *copyright*, which is a legal concept that grants the creator of an original work the exclusive rights to its use and distribution (WIPO 2011). The term *work* includes a wide range of intellectual creations, such as text, photographs, diagrams, maps, movies, etc (Hagedorn et al. 2011). Copyright protects works that are original, individual and new regarding their form of presentation (Agosti and Egloff 2009). Data ownership indicates both responsibility and control over the information. The control of information includes *the ability to access, create, modify, package, derive benefit from, sell or remove data, but also the right to assign these access privileges to others* (Loshin 2002). Copyright gives the owner the exclusive right to reproduce its work, distribute it, communicate it to the public, create derivative versions, and transfer the rights to others (WIPO 1979). The data owner is the person or legal entity possessing the IP rights resulting from the act of creating a data record. The data provider, which may or may not be identical with the data owner, is the person or legal entity submitting the data to LWG RI and having the responsibility for the contents of the dataset. For example, the owner for data resulting from a project is the institution conducting the project, the dean or the director of the institution, while the principal investigator of the project who assembled the data can act as the data provider.

Usually anyone creating an original work automatically holds the copyright on it, which normally expires 70 years after the death of the creator (EU Directive 2006/116/EC). European copyright legislation is well aware of the fact that copyright may present a barrier to scientific work, therefore excep­tions and limitations to the respective copyrights are applied in such cases. For example, while databases as a whole may be protected by copyright law, if they “by reason of the selection or arrangement of their contents, constitute the author's own intellectual creation" (EU Directive 96/9/EC), the single data points, measurements and observations within the database cannot be copyrighted: “The copyright protection of databases provided for by this Directive shall not extend to their contents and shall be without prejudice to any rights subsisting in those contents themselves” (EU Directive 96/9/EC). The Copyright Directive (EU-Directive 2001/29/EC) allows EU Member States to provide exceptions or limitations to the rights of reproduction and communication to the public when a work is used "*for the sole purpose of illustration for teaching or scientific research, as long as the source, including the author's name, is indicated, unless this turns out to be impossible*". However, as suggested by the Green Paper on Copyright in the Knowledge Economy published in 2008, different treatments of the same act in the different countries may lead to legal uncertainty regarding permissions and exceptions, especially when research is carried out within transnational projects.

LifeWatchGreece RI uses Creative Commons (CC) as a legal instrument to define the usage rights of the data. Creative Commons is legally binding, simple to use, globally accepted and its licenses are both human and machine-readable, the latter being especially important in the digital era. *Licenses* are legally binding texts that define what can be done with a work by a third party and they can change the content of the work from “all rights reserved” (copyright) to “some rights reserved”. The LifeWatchGreece RI data are released under two different conditions: 1) CC-Attribution (CC-BY): “*You must give appropriate credit, provide a link to the license, and indicate if changes were made. You may do so in any reasonable manner, but not in any way that suggests the licensor endorses you or your use*”; and 2) CC-Zero (CC0, waiver): “*The person who associates a work with this deed has dedicated the work to the public domain by waiving all of his/her rights to the work worldwide under copyright law, including all related and neighboring rights, to the extent allowed by law. You can copy, modify, distribute and perform the work, even for commercial purposes, all without asking permission*.” A waiver is a declaration for complete copyright removal, meaning that the content will have “no rights reserved” and will be released into the public domain. In that case, the original owner abnegates all his/her rights to the data, but also the responsibility, and becomes a “donor”. The users of CC0 data should make the appropriate attribution to the data donor for the work done and released as free to be used by the society.

The Creative Commons organisation does not recommend the use of “non-commercial”, “no derivatives” and “share alike” licensing types for licensing scientific data, therefore LifeWatchGreece RI does not offer them. The distinction between commercial and non-commercial research is not applicable, useful or even possible (Egloff et al. 2014, Hagedorn et al. 2011). The products or services of a research institute can be commercialised and used for profit earning either by the institute or by other third parties. Regulations that restrict exceptions and limitations to the re-use of works for non-commercial research purposes could misjudge the reality of science (Egloff et al. 2014).

All the data submitted to LifeWatchGreece RI can be subjected to an embargo period determined by the data owner/provider. This selection applies in cases when the data owner/provider requests a sufficient time period for exploiting the data and publishing the results before making them publicly available. The maximum time for an embargo can be 5 years; however, this time can be renewed repetitively until the copyright expires (i.e. 70 years after the owner’s death). If no embargo renewal is requested the data will automatically be released under the specified CC-license or waiver. During an embargo period, only metadata that describe the dataset will be available online.

## General Principles of the LifeWatchGreece﻿ RI

Biodiversity data accessible via the LifeWatchGreece RI are openly and universally available to all potential users within the terms and conditions that the data owner/provider has identified for his/her data under the framework of the LifeWatchGreece RI Data Sharing Agreement (available in the LWG RI website). In general, LifeWatchGreece RI adopts the following Canadensys norms which are not a legal document but in the form of simple instructions they describe the general principles for data publication, sharing and fair use:

1. **Give credit where credit is due**: always cite the data you are using
2. **Be responsible**: data are published to allow anyone to better study and understand the world around us, so do not use the data in any way that is unlawful, harmful or misleading. Indicate clearly any changes you might make to the original dataset.
3. **Share knowledge**: inform LWG RI if you have used the data, inform the data owner/provider if you have comments about the data, if you notice errors, or if you need more information. All contact details are included in the metadata of the dataset.
4. **Respect the data license**: respect the data license or waiver under which the data have been published as indicated in the metadata. Do not remove the public domain mark (CC-Zero) or provide misleading information about the copyright status.

## Rights and duties of the data providers/owners

The data providers/owners retain some rights to their data according to the CC license they have selected when submitting their data. Unless data are released into the public domain with a waiver (CC-Zero), the data are considered as the intellectual property of the data owner/provider. Copyright therefore remains with the data owner/provider. They also have the right to choose an embargo time, although this right should be exercised with diligence and without eventually hampering the use of the datasets. They are able to change their license type, from CC-By to CC-Zero; however, waivers cannot be changed. The data providers/owners also have the right to withdraw a published dataset from the LifeWatchGreece RI if this action is well justified (i.e. the dataset will not be visible to the public), however in this case the data will remain in the system for archival. Even if a dataset is withdrawn, its metadata will remain always public and visible.

The data providers/owners have the duty to make reasonable efforts to ensure that the data served are accurate. They must provide a required minimum subset of metadata along with the dataset, which will always be publicly available under a CC-Zero waiver. The data providers (if different from the data owners) must provide details regarding the identity of the data owner and they must warrant that they have made the necessary agreements with the original data owner that the data can be made available through the LWG RI. The data providers/owners have the responsibility to restrict access to sensitive data, such as the precise localities of endangered species, key-strategist species with vulnerable populations, biota with high blue growth potential, etc. The data providers/owners have to choose one of the predefined CC licenses or a waiver for the dataset. The data providers/owners explicitly authorize the maintenance, reproduction, distribution, availability and re-use of the data within the LWG RI and other relevant databases (e.g. GBIF, MedOBIS, OBIS). They have to collaborate with the data management team of LWG RI to maintain and manage their datasets and to improve their quality where possible. Versioning is supported for changes in data files and/or metadata.

## Rights, duties and restrictions of ﻿the LifeWatchGreece RI

The LWG RI team has the right to annotate datasets and to mark inconsistencies, where appropriate, in order to ensure data quality. However, no data values can be replaced or corrected without retaining the original data value and without consent by the data owner/provider. Data can be disseminated through other relevant databases (e.g. GBIF, MedOBIS, OBIS) using the same licenses and conditions as signed in the LWG Data Sharing Agreement. LWG RI has the right to reformat the data if necessary in order to make them compliant with international standards for integration with other datasets, infrastructures and storage systems.

LWG RI has the duty to ensure the smooth integration of datasets into the LWG RI and is responsible for the technical aspects of data publication. The LWG RI data managers have to perform data quality control and to provide quality assurance annotation before data publication. They have to create a citation (including authors, title and size of dataset) for each dataset according to the GBIF data publishing guidelines and the Joint Declaration of Data Citation Principles. LWG RI needs to work in close collaboration with the data owner/provider in order to assemble and publish the relevant metadata. They must conceal and protect sensitive data in accordance with the data owner/provider. They need to inform the data owners/providers about the available license options, their rights and duties, the approaching of an embargo expiration, as well as the options for its renewal. They should encourage and facilitate free and accessible data sharing and reusability by assigning default open data licenses to both metadata and complimentary files, but they have to allow the application of terms of use and restrictions when needed. LWG RI needs to ensure that the licenses and embargos are not violated (data protection) by the technical infrastructure and that the original dataset is not lost or any original values are overridden. They have to keep track of the data usage by using a tracking metrics system and provide the data owner/provider with this information upon request. They should assist the data owner/provider in data versioning, assignment of a DOI and tracking citations of datasets through literature. Also, they need to ensure that the data owner is properly acknowledged and to inform the data owner/provider in case the terms of use are violated.

LWG RI does not assert any intellectual property rights in the data made available through its portal. The ownership of the data belongs to the data owner/provider, unless the data are released under a waiver (CC-Zero) to the public domain. LWG RI, its employees and its contractors are not liable or responsible for the data contents or their use, or for any loss, damage, claim, cost or expense however this may arise from a third party's inability to properly use the LWG RI. No financial claim can be made by any of the parties concerning the submission, publication, curation or subsequent use of data and datasets. The use of LWG RI always takes place on the basis that the accuracy and reliability of any data hosted are under the responsibility of the data owner/provider, unless the data are provided under the CC-Zero license for which no such responsibility exists.

## Conclusions

The EU is moving towards the deployment of an open knowledge management system and LWG RI could offer a useful contribution to this direction regarding the biodiversity field. The current legal situation can be considered as unsatisfactory due to the existing inconsistencies between the different countries (Egloff et al. 2014). The international collaboration and the open exchange of knowledge can only be benefited by the unification of exceptions and limitations and the binding of the EU regulations regarding research purposes (Egloff et al. 2014). Open data will increase the transparency and the overall quality of science, while data integration will increase the opportunities for collaboration, as well as the potential for interdisciplinary research (Penev et al. 2011). The LWG RI and the policies it has implemented during the initial years of its construction and operation are supporting the above mentioned principles and are in full accordance with the current EU priorities, thus aiming at an open data research community in the near future.
